# Association between structural and social determinants of health and cognitive functioning and African Americans in the ARCHES cohort

**DOI:** 10.1002/alz70858_104541

**Published:** 2025-12-26

**Authors:** Jean‐Francois Trani, Yiqi Zhu, Ramkrishna Kumar Singh, Semere Bekena, Ganesh M. Babulal

**Affiliations:** ^1^ Washington University, St. Louis, MO, USA; ^2^ Natiobal conservatory of Arts and Crafts, Paris, NA, France; ^3^ Washington University School of Medicine, Saint Louis, MO, USA; ^4^ University of Johannesburg, Johannesburg, Gauteng Province, South Africa; ^5^ Knight Alzheimer Disease Research Center, St. Louis, MO, USA

## Abstract

**Background:**

Structural and social determinants of health (S/SDOH) are linked to dementia, yet research on Black Americans is limited. This is crucial given the exponential dementia risk among the Black population. This study examined a new S/SDOH composite index based on the multidimensional poverty‐adjusted headcount ratio and explores its association with possible cognitive impairment (PCI) or poor cognitive performance (PCP) among Black Americans.

**Methods:**

Participants were aged 45 or older, self‐identified as Black or African American, and lived in the greater St. Louis, Missouri area. They were cognitively normal (CN) based on the Montreal Cognitive Assessment (MOCA). The S/SDOH‐CI used 37 deprivation indicators from the National Institute on Aging Health Disparities Research Framework, covering environmental, sociocultural, and behavioral dimensions. Multivariate regression models were used to investigate associations between dementia and S/SDOH‐CI.

**Results:**

Among 314 Black adults, 106 (33.8%) had PCI, and 48 (15.3%) had PCP. A higher proportion of those with PCI or PCP were deprived on 11 indicators (22.9% and 23.4%, respectively), compared to CN adults (13% and 15.1%). The difference in S/SDOH‐CI between Black adults with PCI/PCP and CN was 62.4% and 43.9%, respectively. Key contributors of S/SDOH‐CI included social factors, detrimental health behaviors, limited education, healthcare barriers, and psychological factors. The S/SDOH‐CI was strongly associated with PCI (odds ratio [OR], 11.48; 95%CI 2.08‐65.00) and PCP (OR,13.84; 95%CI 1.27‐152.07) after adjusting for sex, age, and marital status. The risk was higher for Black adults over 65 (OR, 1.90; 95%CI 1.14‐3.22 for PCI) and (OR 6.49; 95%CI 2.84‐16.85 for PCP).

**Conclusion:**

By employing ecological analysis, we constructed a general model of dementia while also illustrating how different socioeconomic levels, as measured by neighborhood‐level deprivation via the ADI, shaped distinct experiences and perceptions of the system contributing to dementia risk. Our findings suggest that improving S/SDOH early in life for Black Americans through targeted public policies could reduce dementia risk later. This includes promoting equitable access to education and healthcare and implementing programs to reduce material hardship. The results also support policies that address structural racism and its impact on the health and well‐being of Black Americans.

